# Confocal Laser Endomicroscopy for the Diagnosis of Urothelial Carcinoma in the Bladder and the Upper Urinary Tract: Protocols for Two Prospective Explorative Studies

**DOI:** 10.2196/resprot.8862

**Published:** 2018-02-07

**Authors:** Esmee IML Liem, Jan Erik Freund, Joyce Baard, D Martijn de Bruin, M Pilar Laguna Pes, C Dilara Savci-Heijink, Ton G van Leeuwen, Theo M de Reijke, Jean JMCH de la Rosette

**Affiliations:** ^1^ Department of Urology Academic Medical Center University of Amsterdam Amsterdam Netherlands; ^2^ Department of Biomedical Engineering & Physics Academic Medical Center University of Amsterdam Amsterdam Netherlands; ^3^ Academic Medical Center University of Amsterdam Amsterdam Netherlands; ^4^ Department of Pathology Academic Medical Center University of Amsterdam Amsterdam Netherlands

**Keywords:** confocal laser endomicroscopy, optical imaging, urothelial carcinoma, nonmuscle invasive bladder carcinoma, upper urinary tract carcinoma, urothelial cancer grading, transurethral resection bladder tumor, ureterorenoscopy, nephroureterectomy, segmental ureter resection, biopsy

## Abstract

**Background:**

Visual confirmation of a suspicious lesion in the urinary tract is a major corner stone in diagnosing urothelial carcinoma. However, during cystoscopy (for bladder tumors) and ureterorenoscopy (for tumors of the upper urinary tract) no real-time histopathologic information can be obtained. Confocal laser endomicroscopy (CLE) is an optical imaging technique that allows for in vivo high-resolution imaging and may allow real-time tumor grading of urothelial lesions.

**Objective:**

The primary objective of both studies is to develop descriptive criteria for in vivo CLE images of urothelial carcinoma (low-grade, high-grade, carcinoma in situ) and normal urothelium by comparing CLE images with corresponding histopathology.

**Methods:**

In these two prospective clinical trials, CLE imaging will be performed of suspicious lesions and normal tissue in the urinary tract during surgery, prior to resection or biopsy. In the bladder study, CLE will be performed in 60 patients using the Cystoflex UHD-R probe. In the upper urinary tract study, CLE will be performed in 25 patients during ureterorenoscopy, who will undergo radical treatment (nephroureterectomy or segmental ureter resection) thereafter. All CLE images will be analyzed frame by frame by three independent, blinded observers. Histopathology and CLE-based diagnosis of the lesions will be evaluated. Both studies comply with the IDEAL stage 2b recommendations.

**Results:**

Presently, recruitment of patients is ongoing in both studies. Results and outcomes are expected in 2018.

**Conclusions:**

For development of CLE-based diagnosis of urothelial carcinoma in the bladder and the upper urinary tract, a structured conduct of research is required. This study will provide more insight in tissue-specific CLE criteria for real-time tumor grading of urothelial carcinoma.

**Trial Registration:**

Confocal Laser Endomicroscopy: ClinicalTrials.gov NCT03013894; https://clinicaltrials.gov /ct2/show/NCT03013894?term=NCT03013894&rank=1 (Archived by WebCite at http://www.webcitation.org/6wiPZ378I); and Dutch Central Committee on Research Involving Human Subjects NL55537.018.15; https://www.toetsingonline.nl /to/ccmo_search.nsf/fABRpop?readform&unids=6B72AE6EB0FC3C2FC125821F001B45C6 (Archived by WebCite at http://www.webcitation.org/6wwJQvqWh). Confocal Laser Endomicroscopy in the upper urinary tract: ClinicalTrials.gov NCT03013920; https://clinicaltrials.gov/ct2/show/NCT03013920? term=NCT03013920&rank=1 (Archived by WebCite at http://www.webcitation.org/6wiPkjyt0); and Dutch Central Committee on Research Involving Human Subjects NL52989.018.16; https://www.toetsingonline.nl/to/ccmo_search.nsf/fABRpop?readform&unids=D27C9C3E5755CFECC12581690016779F (Archived by WebCite at http://www.webcitation.org/6wvy8R44C).

## Introduction

Urothelial carcinoma is the most common malignancy of the urinary tract. The majority of these tumors are located in the bladder and only 5% are located in the upper urinary tract [1]. For bladder cancer, direct visualization of the urothelium with white light cystoscopy (WLC) is the cornerstone for diagnosis and follow-up. Despite its effectiveness and established role, WLC has limitations, such as, its diagnostic accuracy, especially for carcinoma in situ (CIS) [2]. Histopathologic grading and staging of urothelial carcinoma are essential for diagnosis, prognosis and choice of therapy. However, real-time histopathologic assessment is lacking during cystoscopy in an outpatient setting as well as in the operating theatre. For upper tract urothelial carcinoma (UTUC), ureterorenoscopy (URS) with endoscopic biopsies of suspicious areas is considered the diagnostic standard. Also for UTUC, histopathologic evaluation is essential for prognosis and treatment selection as endoscopic treatment is reserved for low grade tumors only [3]. Similar to the diagnostics of bladder cancer, real-time histopathologic assessment is lacking during URS. The use of optical imaging techniques, such as Confocal Laser Endomicroscopy (CLE), may enable real-time optical biopsies to overcome these limitations for bladder cancer and UTUC evaluation.

CLE is a fiber optic probe–based imaging technique that enables real-time in vivo optical sectioning of tissue. The Cellvizio CLE system uses a low-energy 488 nm laser source. The presence of a pinhole limits the detection to in-focus backscattered fluorescent light, enabling high resolution imaging in a single horizontal plane. CLE imaging requires the administration of a fluorescent contrast agent. The most commonly used fluorescent dye is fluorescein. Topical or intravenous application of fluorescein stains the extracellular matrix and enables visualization of the cellular microarchitecture and morphology. Tissue types can be differentiated based on these specific cellular features. For in vivo endoscopic CLE imaging, various sized probes with different image properties are commercially available. CLE was initially applied for in vivo imaging in the gastrointestinal tract and applications in pulmonology are explored [4-6]. In urology, CLE was first examined in the bladder. It seemed feasible to differentiate between normal mucosa and urothelial carcinoma using CLE imaging in a pilot study. Based on histopathology from resected bladder tumors, CLE criteria to differentiate between normal bladder tissue, low-grade, and high-grade bladder tumors were proposed.[7,8] These criteria have also been suggested for the upper urinary tract as the histologic morphology and microarchitecture are alike. However, a CLE probe with different imaging properties is used in the upper urinary tract and only small patient cohorts have been evaluated [9,10].

The development of CLE towards real-time optical biopsies of urothelial carcinoma may lead to advances in diagnosis and prognosis and may affect the cost-benefit of the disease management. Currently, bladder cancer is the most expensive solid malignancy per patient. The high recurrence rate of early stage tumors with long-term survival and adjuvant treatments with close follow-up results in high costs [11,12]. Even though laser fulguration of low-risk tumors has been performed in outpatient settings, it is not widely used due to the lack of histologic confirmation and, therewith, the risk of inadequate treatment [13,14]. Immediate evaluation of tumor grade with CLE could potentially increase the application of laser fulguration and enable treatment of real-time confirmed low-grade tumors in an outpatient setting. Application of laser fulguration in an outpatient setting would lead to an increase in availability of treatment of low-grade bladder cancer and reduction of medical costs. Potentially, CLE may also allow for real-time evaluation of surgical radicality and, therewith, reduce recurrence rates in urothelial cancer. In the upper urinary tract, CLE has the potential to improve the diagnostic approach for UTUC.

Accurate staging and grading of UTUC remains challenging. Visual white light assessment of UTUC grade during URS is inaccurate in approximately 30% of the cases [15]. Moreover, the restricted anatomic space of the upper urinary tract and the subsequent miniaturization of tissue-harvesting instruments limit the yield of ureteroscopic biopsies. The diagnostic yield and the diagnostic accuracy for tumor stage of endoscopic biopsies are limited [16,17]. However, tumor grade from endoscopic biopsies may indicate tumor stage [18,19]. As such, tumor grade from endoscopic biopsies is a major decisive factor for endoscopic treatment eligibility. Though, in 69-90% of endoscopic biopsies, tumor grade is in concordance with the histopathologic grade from radical resection [17,18,20,21]. Moreover, endoscopic biopsies hold a risk of complications.

CLE may overcome such diagnostic limitations for tumor grade assessment. Optical real-time assessment of tissue type and tumor grade could aid perioperative clinical decision-making. Histologic assessment without tissue biopsies could prevent possible impaired endoscopic vision after biopsies during URS. Additionally, the digital data from CLE imaging allows for real-time computer aided diagnosis with software, augmenting the practical and diagnostic value of optical imaging techniques. Further exploration of different optical imaging modalities for tumor diagnosis may lead to multimodal optical biopsies for real-time tumor grading and staging, possibly replacing tissue biopsies in future. However, a structured conduct of research is required to guide us towards optical biopsies. The aim of these two study protocols is to contribute to the development of essential knowledge for CLE-based diagnosis of urothelial carcinoma in the bladder and the upper urinary tract. In this paper, we describe two study protocols for CLE in the urinary tract together as both protocols share many methodological and disease-specific similarities.

## Methods

### Study Objectives

The primary objective of both studies is to develop descriptive criteria for in vivo CLE images of urothelial carcinoma (low-grade, high-grade, CIS) and normal urothelium by comparing CLE images with corresponding histopathology.

Secondary objectives are to develop a CLE image atlas of the urinary tract, to assess the technical feasibility and procedure-related adverse events, to assess CLE image quality, to qualitatively evaluate CLE images, to preliminarily assess the diagnostic yield, and to establish an estimation of the diagnostic accuracy of CLE-based diagnosis in comparison with histopathology and to assess inter-observer agreement.

### Study Design

Approval of the local medical ethical committee was obtained for both study protocols (registry number: NL55537.018.15 and NL52989.018.16). Both studies are prospective, single-centre, in vivo, observational human studies to assess CLE features of normal urothelium and urothelial carcinoma (low-grade, high-grade or CIS) in the bladder and in the upper urinary tract. Both explorative studies are in agreement with the IDEAL stage 2b recommendations [22]. The two study protocols differ mainly in the location of urothelial carcinoma, and subsequently, the surgical approach, the type of CLE probe, the administration of fluorescein, and the reference standard. Differences in protocols are explained separately and listed in Table 1.

**Figure 1:**
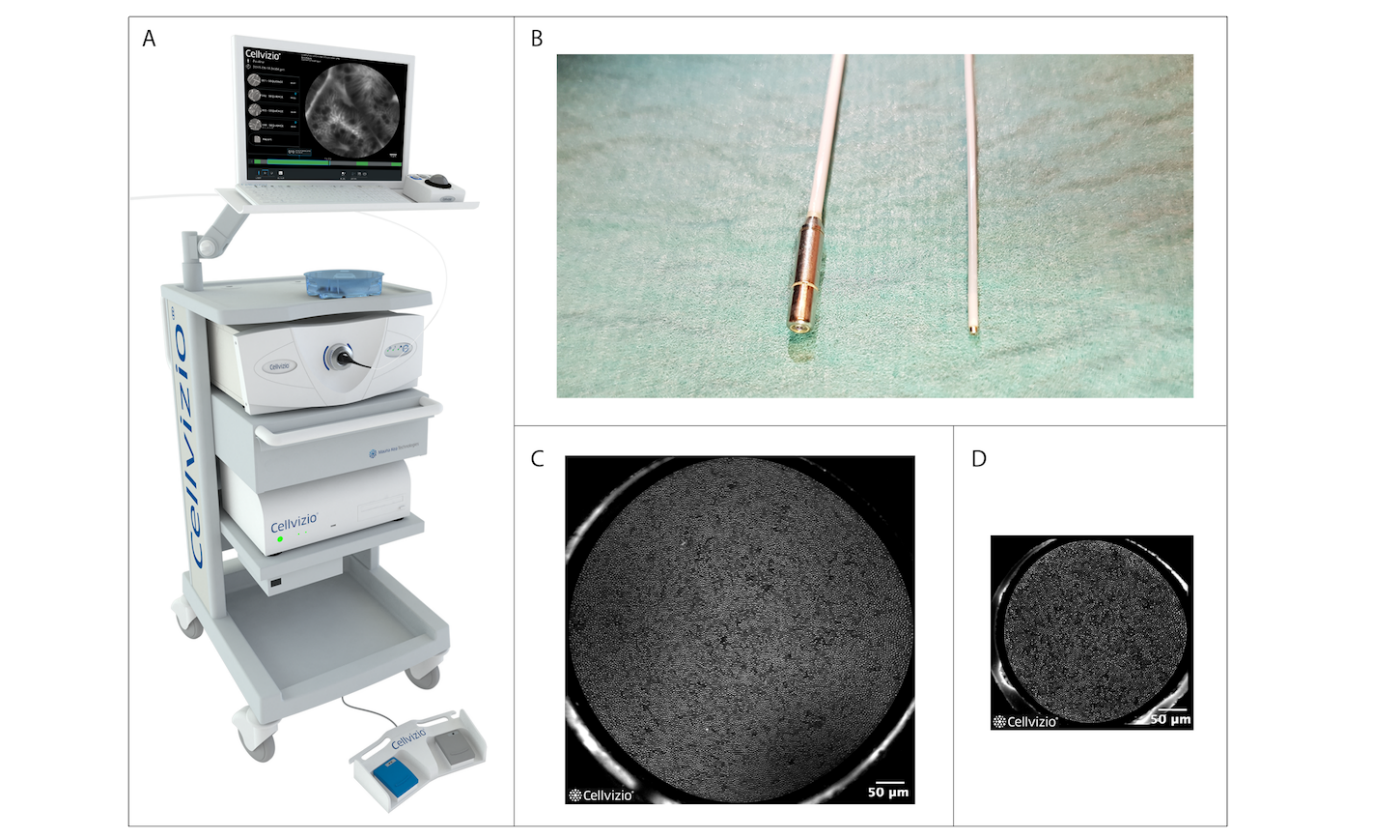
Cellvizio CLE (Confocal laser endomicroscopy) system and the probes used for the urinary tract. (A) Cellvizio CLE system; (B) The two different probes used in the urinary tract. On the left the Cystoflex UHD-R probe with a diameter of 2.6 mm, which is used in the bladder. On the right the Uroflex-B probe with a diameter of 0.85 mm, which is used in the upper urinary tract; (C) RAW image of the Cystoflex UHD-R probe displaying the single fibers; (D) RAW image of the Uroflex-B probe displaying the single fibers.

For both studies, CLE images are recorded with a fiber optic probe–based system (Cellvizio 100 series, Mauna Kea Technologies, Paris, France). The Cystoflex UHD-R probe of 8.4 Fr (Mauna Kea Technologies, Paris, France) is used for CLE imaging in the bladder. The Uroflex-B probe of 2.7 Fr (Mauna Kea Technologies, Paris, France) is used in the upper urinary tract. The smaller Uroflex-B probe contains less optical fibers and, therewith, a lower image resolution compared to the Cystoflex UHD-R probe. Both forward-looking probes are illustrated in Table 1 and Figure 1.

CLE imaging requires the application of a fluorescent contrast agent. Fluorescein (fluoresceinedisodium, Fresenius Kabi, Zeist, The Netherlands) is a non-toxic and commonly used fluorescent dye for CLE imaging [23]. It stains the extracellular matrix and is administered topically prior to CLE imaging during the surgical procedure.

In both studies, patients will undergo in-vivo CLE imaging during surgery, prior to resection or biopsy of suspicious areas for standard histopathologic assessment. The probes are introduced through the working channel of the standard endoscopes. In the bladder, a Karl Storz cystoscope of 22 Fr with 0° optics is used for CLE imaging. Transurethral resection is subsequently performed with a Karl Storz or Olympus resectoscope of 26 Fr. For CLE imaging of the upper urinary tract, a flexible digital ureterorenoscope of 8.5 Fr is used (Karl Storz Flex Xc or Olympus V2). After placing the probe in direct contact with the region of interest, image sequences of 8–12 frames per second of the real-time visualization of the cellular microarchitecture are recorded (Multimedia Appendices 1 and 2). In general, recording is conducted in both protocols twice for 1 minute of the region of interest. In case of multiple regions of interest, multiple CLE recordings are performed. At a later stage, recorded CLE images will be analyzed independently by three blinded observers and compared to the corresponding histopathology. For CLE imaging in the bladder, the reference standard for comparison of histopathology will be the specimen of the en-bloc resected lesion. For the upper urinary tract, the reference standard will be the specimen of the radical resection (radical nephroureterectomy [RNU] or segmental ureter resection [SU]). Histopathology analysis is performed according to the standard clinical protocol and is performed by a specialized uropathologist (CDSH), who is blinded for the CLE images. A follow-up of 30 days is considered to register any adverse events following the study procedure.

### Population and Sample Size

Patients eligible for either study are >18 years with a suspicious lesion in the urinary tract, scheduled for either transurethral resection (TURB) (lower urinary tract) or diagnostic URS (upper urinary tract). The main exclusion criteria are fluorescein allergy and pregnancy (Table 1). All patients will be recruited in the AMC Hospital (Amsterdam, The Netherlands), and all study procedures will be performed in this institution. Patients will be informed about the study in oral and written form. Patient inclusion is confirmed by signing written informed consent. Patients will only be included in one study at the time. A total of 60 consecutive evaluable patients with bladder tumors or suspicion of CIS will be included in the bladder cancer study. Based on prevalence, we expect to include 32 low-grade, 22 high-grade, and 6 CIS lesions. For the upper urinary tract study, 25 patients with UTUC that will undergo a radical treatment after the diagnostic URS will be included. However, the indication for radical treatment is determined after diagnostic URS. In general, about one-third of the UTUC patients are treated with radical surgery in our centre. Therefore, we expect to include 70 consecutive UTUC patients to reach the total number of 25 patients who will undergo radical treatment. Considering the possible selection bias for radical treatment, we expect to include 10 low-grade, 12 high-grade, and 3 CIS lesions. These sample sizes are based on prior publications and comply with the IDEAL recommendations for explorative studies [7,9,22].

**Table 1:** Differences between the two study protocols. CIS: carcinoma in situ; CLE: Confocal Laser Endomicroscopy; RNU: radical nephroureterectomy; SU: segmental ureter resection, TURB: transurethral resection bladder tumor; URS: ureterorenoscope; UTUC: upper tract urothelial carcinoma.

Variables	CLE bladder study	CLE upper urinary tract study
Population	60 consecutive patients with primary or recurrent bladder tumor	25 patients with UTUC that will undergo a RNU or SU after diagnostic URS with CLE imaging
Inclusion criteria	bladder tumor or possible CIS scheduled for TURB signed informed consent	suspicion of UTUC scheduled for diagnostic URS signed informed consent
Exclusion criteria	allergy for fluorescein possible pregnancy or lactating women	allergy for fluorescein possible pregnancy or lactating women Patients not eligible for RNU
Urologic instruments at use	Karl Storz 22 Fr cystoscope with 0° optics for CLE imaging, and Karl Storz or Olympus 26 Fr resectoscope for transurethral resection	Karl Storz Flex Xc or Olympus V2 8.5 Fr flexible digital ureterorenoscope
Contrast agent	Topical application of 300-400 mL of 0.1% fluorescein via Foley catheter and left indwelling for 5 minutes	Topical application of 0.5 mL 2.5% fluorescein via working channel for immediate imaging
CLE probe	Cystoflex UHD-R diameter 2.6 mm lateral resolution 1 µm field of view 240 µm imaging depth 50-65 µm	Uroflex-B diameter 0.85 mm lateral resolution 3.5 µm field of view 320 µm imaging depth 40-70 µm
Histopathologic reference standard	En-bloc resected bladder tumor	RNU or SU

### Study procedures

#### Protocol for Confocal Laser Endomicroscopy in the Bladder

In the operating theatre prior to the TURB under general or regional anaesthesia, visual inspection with WLC and image enhancement modalities (narrow band imaging or Image1S) will be performed. At least one suspicious lesion (papillary or flat) and one region of normal appearing bladder tissue will be selected for CLE imaging. The regions of interest will be marked laterally with the cautery electrode for topographic matching. After marking the regions of interest, 300-400 mL of fluorescein (0.1% fluorescein diluted in saline) will be administered intravesically using an indwelling catheter. After instillation of the fluorescein for 5 minutes, the bladder will then be emptied and excessive fluorescein will be rinsed out. The Cystoflex UHD-R probe will be introduced through the working channel of a 22 Fr Karl Storz cystoscope with 0° optics. After placing the probe into direct contact and perpendicular to the tissue, CLE images will be recorded twice for approximately 1 minute of each marked region. After CLE imaging, the tumor will be resected en-bloc and a small chip of the marked normal urothelium will be resected for histopathologic matching. Transurethral resection is performed with a Karl Storz or Olympus resectoscope of 26 Fr.

#### Protocol for Confocal Laser Endomicroscopy in the Upper Urinary Tract

The complete ureter and renal pelvis are inspected with WLC and image enhancement modalities (narrow band imaging or Image1S) during standard flexible digital ureterorenoscopy under general anaesthesia to identify regions of interest. Only in case of visually confirmed upper tract tumors during URS, study-related activities will be performed. If regions of interest are identified, the region that is most easily accessible for endoscopic biopsies is selected for CLE imaging. Fluorescein (0.5 mL of 2.5% fluorescein diluted in saline) is administered through the working channel. The Uroflex-B probe is introduced via the working channel of the 8.5 Fr flexible digital ureteroscope (Karl Storz Flex Xc or Olympus V2) and placed into direct contact with the region of interest for immediate CLE imaging. Each region of interest is imaged twice for approximately 1 minute with CLE. After imaging, endoscopic biopsies for the standard diagnostic process are taken from the same location. Depending on the histopathologic diagnosis, the indication for a radical treatment will be determined.

### Data Analysis

**Figure 2:**
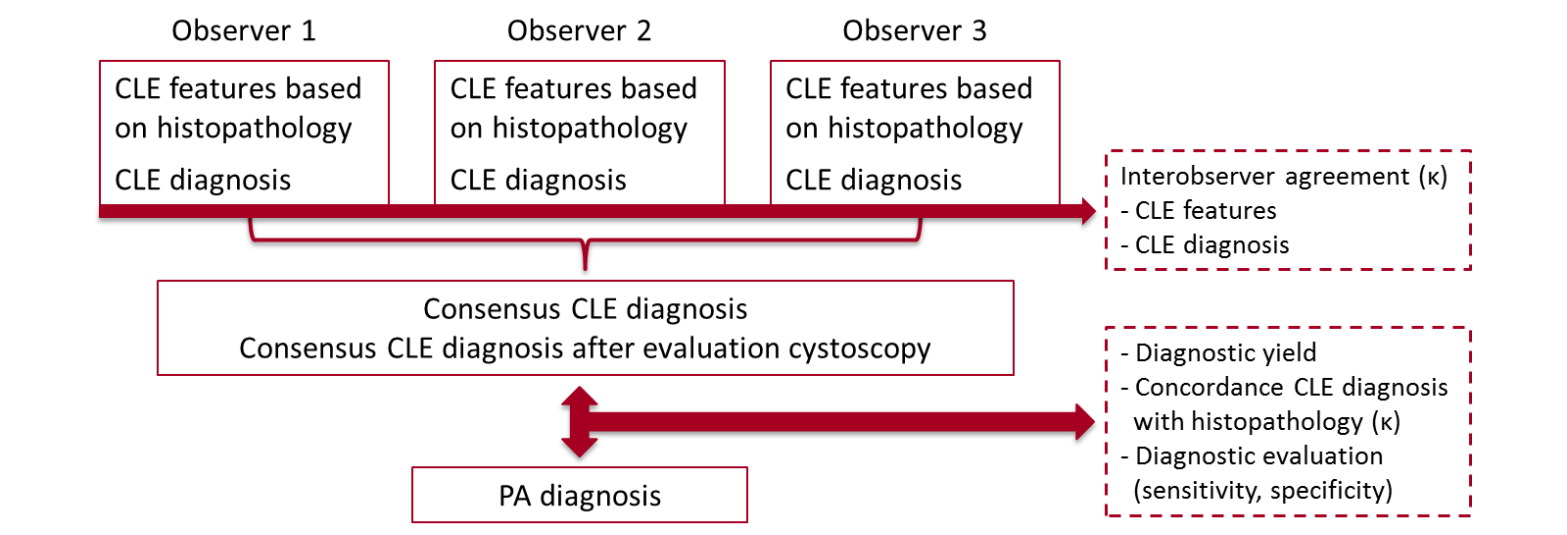
A schematic overview of the data analysis plan. CLE: confocal laser endomicroscopy; PA: histopathology.

The method of analysis is identical for both study protocols. Demographic- and disease- specific characteristics of the study populations (eg, age, sex, medical history of urothelial carcinoma, tumor focalitiy, tumor location, and tumor size) will be collected. Three blinded observers, consisting of two researchers (EL & JF) and one uropathologist (CDSH), will independently analyze CLE images frame by frame in an offline setting with the Cellvizio Viewer software (Mauna Kea Technologies, Paris, France). Modified criteria for CLE image evaluation will be used for analysis (Table 2) [8]. The CLE image quality per region of interest will be scored on a Likert scale as poor, fair, or good. Image quality will be used for qualitative evaluation of the technique and for subgroup analysis. Based on histologic features, for each region of interest, a CLE-based diagnosis will be made by each observer as benign urothelium or urothelial carcinoma. The diagnosis of urothelial carcinoma is subdivided in low-grade or high-grade urothelial carcinoma (WHO 2004 classification), CIS, or as inconclusive. An inconclusive CLE diagnosis is defined as poor image quality where CLE features cannot be assessed. After individual analysis, a consensus will be reached for the CLE-based diagnosis by all three observers for each region of interest. The appropriateness of consensus of the CLE-based diagnosis is evaluated by viewing the endoscopic images. After determining CLE-based diagnosis and consensus, CLE images will be compared to the corresponding histopathology specimen (either en-bloc resected bladder tumor or RNU/SU specimen) for evaluation of the concordance of CLE-based diagnosis with histopathologic diagnosis. Differences between diagnosed groups will be analyzed with a chi-square test. For an initial evaluation of the diagnostic value, sensitivity and specificity will be calculated based on a 3x3 table where CIS is classified as high-grade tumor. Proportions of specific agreement and Fleiss Kappa analysis will be used for interobserver agreement of CLE-based diagnosis. A schematic overview of the data analysis is displayed in Figure 2.

**Table 2:** Modified confocal laser endomicroscopy (CLE) image characteristics and their variables for analysis.

CLE feature	Variables
Papillary aspect	Present or not present
Polarity of cells	Present or not present
Organisation of cells	Organized or disorganized
Cohesiveness of cells	Cohesive or discohesive
Cellular morphology	Monomorph or pleiomorph
Definition of cell borders	Distinct or indistinct
Vasculature	Capillary network, fibrovascular stalk, or large vessels

### Safety

The investigators will monitor patient safety. They can withdraw a patient from the study for medical reasons. In accordance to section 10, subsection 4, of the “Wet Medisch-Wetenschappelijk Onderzoek met Mensen” (medical research involving human subjects act in The Netherlands), the investigators will suspend the study if there is sufficient ground that continuation of the study will jeopardise patient health or safety. The investigators will notify the accredited Institutional Review Board if this is the case. In case of an adverse event or serious adverse event, the responsible authorities will be informed.

### Risks and Benefits

There are no direct benefits for patients participating in these two studies. In the future, however, the results of these studies may be important for patients diagnosed with a tumor in the urinary tract. All patients participating in both studies are scheduled for standard treatment of tumor(s) of the urinary tract; either TURB or URS with endoscopic biopsies. CLE is a minimally invasive imaging technique that can be performed in conjunction with conventional endoscopic treatment. Previous studies of CLE combined with topical administration of fluorescein have proven to be safe [7,24]. Fluorescein is a commonly used fluorescent dye, and its safety is well established for its use in ophthalmological angiography [25]. In patients not at risk for a previously demonstrated allergic reaction, this dye is safe. Patients with a known allergic reaction to fluorescein cannot participate in this study.

## Results

Presently, recruitment of patients is ongoing in both studies. Results and outcomes are expected in 2018.

Summarized raw data will be made available through publication in an international peer-reviewed medical journal.

## Discussion

CLE is an optical imaging technique that may enable real-time optical biopsies. The exploration of tissue specific CLE criteria is essential for the development towards real-time tumor grading of urothelial lesions. Both trials will provide more insight into CLE features of urothelial carcinoma in the bladder and the upper urinary tract and into its diagnostic value.

The design of the bladder protocol aims for topographic matching of CLE images with the resected specimen. The cauterisation marks placed laterally to the region of interest prior to CLE imaging ensures that imaging and resection is done of the exact topographic tissue. Nonetheless, it will be challenging to create an identical histopathological slide of the resected specimen in exactly the same plane as the imaged tissue. We assume that this approach is the closest approximation for topographic matching without interference in the standard clinical histopathologic process.

The study design of the upper urinary tract protocol will lead to a surplus of study measurements, considering that only about one-third of the UTUC patients will receive radical surgery as treatment. Since mainly patients with high-grade or high-volume low-grade tumor will qualify for radical treatment, selection bias could be a risk. The data acquired of patients who are not suitable for radical treatment enables secondary analysis for the comparison of CLE images with the histology of endoscopic biopsies of the imaged regions of interest. In the current study design, identical topographic matching of CLE images in the upper urinary tract with the specimen of the RNU is limited by the fact that in general the diagnostic URS with CLE imaging is not performed during the same procedure as the radical resection. However, topographic matching is approximated by tumor mapping during URS (mapping and annotation of location, size, and appearance) for identification of lesions during the histopathologic assessment.

As with all new techniques, a learning curve for the handling and image interpretation may be expected for CLE. Besides potential intraobserver variability, the learning process might also influence the CLE image quality of the first cases. We aim to limit the number of users to a minimum number of experienced endourologists to minimize the potential effect of a learning curve.

Despite these limitations, we expect that the results of these trials will contribute to determining the role of CLE imaging for the diagnosis of bladder cancer and UTUC in clinical practice. In the light of the limitations of cystoscopy and URS, CLE holds the potential to enable real-time tumor grading of urothelial carcinoma.

## Appendix

Multimedia Appendix 1 A demonstration of CLE imaging of a bladder tumour. The CLE probe is in direct contact with the bladder tumour for CLE imaging.

Multimedia Appendix 2 A demonstration of CLE imaging of an upper urinary tract tumour. The CLE probe is in direct contact with the tumour for CLE imaging.

## Abbreviations

CIS: carcinoma in situ
CLE: confocal laser endomicroscopy
PA: histopathology
RNU: radical nephroureterectomy
SU: segmental ureter resection
TURB: transurethral resection of bladder tumor(s)
URS: ureterorenoscopy
UTUC: upper tract urothelial carcinoma
WLC: white light cystoscopy

## References

[1] Siegel RL, Miller KD, Jemal A (2017). Cancer Statistics, 2017. CA Cancer J Clin.

[2] Cina S, Epstein JL, Endrizzi JM, Harmon WJ, Seay TM, Schoenberg MP (2001). Correlation of cystoscopic impression with histologic diagnosis of biopsy specimens of the bladder. Hum Pathol.

[3] Rouprêt Morgan, Babjuk M, Compérat Eva, Zigeuner R, Sylvester RJ, Burger M, Cowan NC, Gontero Paolo, Van Rhijn Bas W G, Mostafid A Hugh, Palou J, Shariat SF (2018). European Association of Urology Guidelines on Upper Urinary Tract Urothelial Carcinoma: 2017 Update. Eur Urol.

[4] Dunbar KB, Okolo P 3rd, Montgomery E, Canto MI (2009). Confocal laser endomicroscopy in Barrett's esophagus and endoscopically inapparent Barrett's neoplasia: a prospective, randomized, double-blind, controlled, crossover trial. Gastrointest Endosc.

[5] He XK, Liu D, Sun LM (2016). Diagnostic performance of confocal laser endomicroscopy for optical diagnosis of gastric intestinal metaplasia: a meta-analysis. BMC Gastroenterol.

[6] Wijmans L, d'Hooghe JN, Bonta PI, Annema JT (2017). Optical coherence tomography and confocal laser endomicroscopy in pulmonary diseases. Curr Opin Pulm Med.

[7] Wu K, Liu JJ, Adams W, Sonn GA, Mach KE, Pan Y, Beck AH, Jensen KC, Liao JC (2011). Dynamic real-time microscopy of the urinary tract using confocal laser endomicroscopy. Urology.

[8] Chang TC, Liu JJ, Hsiao ST, Pan Y, Mach KE, Leppert Jt, McKenney JK, Rouse RV, Liao JC (2013). Interobserver agreement of confocal laser endomicroscopy for bladder cancer. J Endourol.

[9] Bui D, Mach KE, Zlatev DV, Rouse RV, Leppert JT, Liao JC (2015). A Pilot Study of In Vivo Confocal Laser Endomicroscopy of Upper Tract Urothelial Carcinoma. J Endourol.

[10] Villa L, Cloutier J, Cotè JF, Salonia A, Montorsi F, Traxer O (2016). Confocal Laser Endomicroscopy in the Management of Endoscopically Treated Upper Urinary Tract Transitional Cell Carcinoma: Preliminary Data. J Endourol.

[11] Botteman MF, Pashos CL, Redaelli A, Laskin B, Hauser R (2003). The health economics of bladder cancer: a comprehensive review of the published literature. Pharmacoeconomics.

[12] Avritscher EB, Cooksley CD, Grossman HB, Sabichi AL, Hamblin L, Dinney CP, Elting LS (2006). Clinical model of lifetime cost of treating bladder cancer and associated complications. Urology.

[13] Fowler CG, Boorman LS (1986). Outpatient treatment of superficial bladder cancer. Lancet.

[14] Wedderburn AW, Ratan P, Birch BR (1999). A prospective trial of flexible cystodiathermy for recurrent transitional cell carcinoma of the bladder. J Urol.

[15] El-Hakim A, Weiss GH, Lee BR, Smith AD (2004). Correlation of ureteroscopic appearance with histologic grade of upper tract transitional cell carcinoma. Urology.

[16] Vashistha V, Shabsigh A, Zynger DL (2013). Utility and diagnostic accuracy of ureteroscopic biopsy in upper tract urothelial carcinoma. Arch Pathol Lab Med.

[17] Cutress ML, Stewart GD, Zakikhani P, Phipps S, Thomas BG, Tolley DA (2012). Ureteroscopic and percutaneous management of upper tract urothelial carcinoma (UTUC): systematic review. BJU Int.

[18] Guarnizo E, Pavlovich CP, Seiba M, Carlson DL, Vaughan ED Jr, Sosa RE (2000). Ureteroscopic biopsy of upper tract urothelial carcinoma: improved diagnostic accuracy and histopathological considerations using a multi-biopsy approach. J Urol.

[19] Brown GA, Matin SF, Busby JE, Dinney CP, Grossman HB, Pettaway CA, Munsell MF, Kamat AM (2007). Ability of clinical grade to predict final pathologic stage in upper urinary tract transitional cell carcinoma: implications for therapy. Urology.

[20] Straub J, Strittmatter F, Karl A, Stief CG, Tritschler S (2013). Ureterorenoscopic biopsy and urinary cytology according to the 2004 WHO classification underestimate tumor grading in upper urinary tract urothelial carcinoma. Urol Oncol.

[21] Williams SK, Denton KJ, Minervini A, Oxley J, Khastigir J, Timoney AG, Keeley FX Jr (2008). Correlation of upper-tract cytology, retrograde pyelography, ureteroscopic appearance, and ureteroscopic biopsy with histologic examination of upper-tract transitional cell carcinoma. J Endourol.

[22] McCulloch P, Altman DG, Campbell WB, Flum DR, Glasziou P, Marshall JC, Nicholl J, Aronson JK, Barkun JS, Blazeby JM, Boutron IC, Campbell WB, Clavien PA, Cook JA, Ergina PL, Feldman LS, Flum DR, Maddern GJ, Nicholl J, Reeves BC, Seiler CM, Strasberg SM, Meakins JL, Ashby D, Black N, Bunker J, Burton M, Campbell M, Chalkidou K, Chalmers I, de Leval M, Deeks J, Ergina PL, Grant A, Gray M, Greenhalgh R, Jenicek M, Kehoe S, Lilford R, Littlejohns P, Loke Y, Madhock R, McPherson K, Meakins J, Rothwell P, Summerskill B, Taggart D, Tekkis P, Thompson M, Treasure T, Trohler U, Vandenbroucke J, Balliol Collaboration (2009). No surgical innovation without evaluation: the IDEAL recommendations. Lancet.

[23] Wallace M, Meining A, Canto MI, Fockens P, Miehlke S, Roesch T, Lightdale CJ, Pohl H, Carr-Locke D, Löhr M, Coron E, Filoche B, Giovannini M, Moreau J, Schmidt C, Kiesslich R (2010). The safety of intravenous fluorescein for confocal laser endomicroscopy in the gastrointestinal tract. Aliment Pharmacol Ther.

[24] Sonn GA, Jones SN, Tarin TV, Du CB, Mach KE, Jensen KC, Liao JC (2009). Optical biopsy of human bladder neoplasia with in vivo confocal laser endomicroscopy. J Urol.

[25] Moosbrugger KA, Sheidow TG (2008). Evaluation of the side effects and image quality during fluorescein angiography comparing 2 mL and 5 mL sodium fluorescein. Can J Ophthalmol.

